# How does disability contribute to deprivation in ageing process: a multidimensional analysis

**DOI:** 10.3389/fpubh.2025.1587613

**Published:** 2025-06-19

**Authors:** Kairan Zhang, Jiayi Wang

**Affiliations:** ^1^School of Political Science and Public Administration, Northwest University of Political Science and Law, Xi’an City, China; ^2^School of Public Administration and Policy, Renmin University of China, Beijing, China

**Keywords:** disability, multidimensional deprivation, ageing, older adults, China

## Abstract

**Background:**

In the context of disability and multidimensional deprivation during the ageing process, this paper aims to assess the net effect of multidimensional deprivation in older adults induced by disability with bidirectional causality controlled.

**Methods:**

Using data from the China Health and Retirement Longitudinal Study 2011–2018, this paper estimated the multidimensional deprivation in terms of economic condition, subjective well-being, and social participation caused by old age disability, which was assessed by a joint identification method, with instrumental variable employed.

**Results:**

The results indicate that (1) the higher the degree of disability, the higher the probability and degree of multidimensional deprivation suffered by the old age. (2) And the level of disability has caused more significant deprivation in subjective well-being and social participation dimensions than economic condition. (3) The effect of multidimensional deprivation induced by disability also varies by urban and rural areas.

**Conclusion:**

More comprehensive health and anti-deprivation policies are needed to accurately identify disability status, prevent associated socio-economic risks, and narrow the urban–rural gap in disability and deprivation.

## 1 Introduction

With the rising life expectancy alongside declining mortality and fertility rates, the number and proportion of older adults have been rapidly increasing. Health problems such as incapability in daily living, cognitive impairment, perceptual decline, and depression have become prominent increasingly with the feature getting older physiologically. It is estimated that there are currently more than 450 million older adults with disability worldwide (1). Meanwhile, due to the cessation of labor, the income of older adults usually decreases significantly, making them vulnerable and placing them at high risk of poverty (2). The above aspects may combine to further affect the social participation of older persons. Overall, the disability and relative deprivation in multidimensions faced by the old age have become global issues affecting social development in the era of ageing.

It should be pointed out that disability and multidimensional deprivation are not only common trends accompanying the ageing process, but also interact and influence each other. On one hand, the older adults with disability may face greater economic risks because of their increased care need and care expenditure (3). And the decline in objective physical abilities may also affect their participation in social activities, as well as their subjective well-being, satisfaction, confidence, etc. (4). These aspects are what it called “multidimensional deprivation.” On the other hand, older adults with relatively disadvantaged socioeconomic status are more likely to be at risk of disability (5, 6).

In this context, recent researches have increasingly focused on disability and multidimensional deprivation among old adults, and mainly include three research areas. The first is measuring of the disability degree in older adults, as well as analyzing the causes and consequences of disability (7, 8). The second is the calculation of multidimensional deprivation taking into account the specificities of older adults (9). The third, is the relationship between disability and multidimensional deprivation. It has been found that the consequences of disability are progressively expanding from economic deprivation caused by increased care cost (10) to non-economic deprivation such as impaired health, limited social participation, etc. (11). And disability has been proved playing a significant role in deprivation duration (12).

However, there are still some limitations in above studies. First, as it has been found that there may be interrelationships between different kinds of capability (13), current measurement for disability from single dimension may cause underestimation or overestimation. Thus, joint identification from multiple dimensions is needed to provide more accurate measurement of the probability, degree and population size of disability. Second, while existing research mainly focusing on the consequences of disability in different dimensions separately, only a few studies estimated the comprehensive consequences of disability from multiple dimensions. Meanwhile, some studies combined dimensions which may be directly affected by disability including economic conditions, social participation, subjective well-being, with dimensions which are not the “direct result” but “improvement practice” of disability such as living facilities (14). This may lead to a biased estimation of the consequences caused by disability when using multidimensional deprivation as outcome variable.

To fill these gaps, this paper aims to achieve two goals. Firstly, to provide more accurate measurement of the disability degree in older adults from the perspective of multiple abilities. Secondly, to accurately estimate the extent and mechanisms by which disability exacerbates multidimensional deprivation in older adults using consequence dimensions more susceptible to disability. By deepening the understanding of the interaction between disability and deprivation, this paper can provide evidence and reference for social policies to improve health and alleviate deprivation in old age.

The rest of this paper is structured as follows. Section 2 provides literature review related to the measurements and consequences of disability. Section 3 describes the data sources, variable measurements, and empirical models. Section 4 presents main results of disability identification, measurement and decomposition of multidimensional deprivation, as well as the impact of disability on multidimensional deprivation. Section 5 discusses interpretation and extrapolation of estimates, and Section 6 provides policy implications, research limitations and prospects. The Appendix provides additional details.

## 2 Literature review

### 2.1 Measurement of disability from multiple indicators

Since the second half of the 20th century, a growing body of literature has focused on the topic of disability, which leads to an enrichment of related concepts and theoretical models (15–17), as well as the evolution of disability measurement, resulting in three generations of assessment tools.

The first generation of assessment tools was dominated by single-ability measures, including ADL (18) and IADL (19). The second generation featured multidimensions consisting of somatic functioning, health care, socioeconomic environment, and life quality, such as the EASY-Care in the U. K. (20), NBA in Germany (21) and ACFI in Australia (22). And the third generation represented by the International Resident, Assessment, Instruments (23), has broken down the barriers of health information between different countries and care institutions, making the data and information accessible, transferable and continuous.

However, constrained by operability, the current assessment of disability in research and practice is still dominated by the first-generation tools. This single-standard approach not only fails to fully identify the difficulties of the old people in other abilities, but may also limit the development of relevant policies and care services due to the underestimation of care needs. Although some studies have incorporated indicators including cognitive impairment, communication, and social participation (24), and some have already focused on the interrelationships between different kinds of disability such as the ADL, IADL, cognitive impairment, etc. (13), the research that truly identifies and assesses disability using the second-generation tool is still quite sparse. Therefore, there is still an urgent need to construct a comprehensive disability measurement that can be put into practice and research.

### 2.2 Multidimensional deprivation as consequence of disability

With the worldwide evolution of economic development and socio-demographic structures, researches on the socio-economic consequences of disability had shown two shifts. First, discussions on the consequences scopes of disability have expanded from single dimension to multiple dimension, i.e., from economic deprivation due to the increased cost of care services (10) to non-economic deprivation, such as impaired health, contrained social participation, etc. (11). Second, the focus on the duration of the disability consequences has expanded from short-term and static deprivation to long-term and chronic deprivation. While earlier studies found that the process of disability has a continuing impact on individuals and families (25), recent studies have further validated the important role of disability in the duration of poverty (12) and its impact on getting rid of poverty and re-impoverishment (26). In addition, existing research has also found some heterogeneous consequences of disability led by the distribution of services, resources, and facilities (9, 27).

Although the above studies provide a theoretical basis for this paper, there are still gaps that need to be further explored. Firstly, previous research has expanded the focus on the dimensions of consequences of disability, recognizing that disability may lead to multiple overlapping risks for older adults (28). However, these researches mainly analyzed the consequences of disability in separate dimensions, making it difficult to estimate the extent of deprivation caused by disability from a holistic and comprehensive perspective.

Secondly, it should be recognized that multidimensional deprivation indicators aimed at providing a comprehensive “measurement” should be distinguished from those as a “consequence” of disability. Current measurement of multidimensional deprivation of the old adults is mainly based on the Multidimensional Poverty Index (MPI) proposed by the United Nations (29), which excludes education dimension and the indicator of child mortality in health dimension, and increases dimensions of social participation and social security (9, 30, 31). However, some of these indicators may be inappropriate if using as consequences of disability. This is because the changes in indicators related to policy and environment are often not the direct consequences of disability status, but rather general improvements accompanying with social development or specific policy practices. Specifically, old-age disability does not change the equipment of water, electricity and gas if the household already has such living conditions. And the old will also not be deprived of their pension and health insurance if they have already participated in such social protections. Comparatively, only indicators that could be directly affected and deprived by the occurrence or aggravation of disability, such as income, consumption, subjective well-being, depression, social activity, etc., should be used as the consequences of disability.

To summarize, the marginal contributions of this paper to existing research lie in two aspects. Firstly, based on an approach from multiple abilities, this paper provides a new perspective and empirical evidence for accurately assessing the disability degree. Secondly, through proper dimensional selection and causal analysis, this paper analyzes and explains how and to what extent the occurrence and aggravation of disability would lead to the multidimensional deprivation faced by old adults.

## 3 Methods

### 3.1 Data and samples

This paper uses China data as the representative case. As the developing country with the largest old population in the world, China had around 46 million older adults living in relative poverty (32), and 52.71 million disabled older adults in 2020 (33). And it is predicted that by 2030, there would be 13.32 million disabled and impoverished older adults in urban areas alone. Overall, the situation of deprivation and disability among older adults will be grim in China (34).

To be specific, the data used for analysis is from 4 waves of China Health and Retirement Longitudinal Study (CHARLS), a longitudinal survey on a representative sample of Chinese residents conducted in 2011, 2013, 2015 and, 2018, covering 12,400 households in 150 counties and 450 communities (villages) of 28 provinces in mainland China. According to the research aim, the participants under 60 years old and those investigated only once were excluded. Finally, a total of 32,926 samples were included.

### 3.2 Variable measurements

#### 3.2.1 Independent variable: disability

The measurement of disability is based on a second-generation assessment tool developed by China. In 2021, the National Healthcare Security Administration (NHSA) issued the “Long-Term Care Disability Rating Assessment Standards (Trial),” which combines the international standards of disability (35, 36) with the demographic characteristics and policy priorities in China.

**Figure 1:**
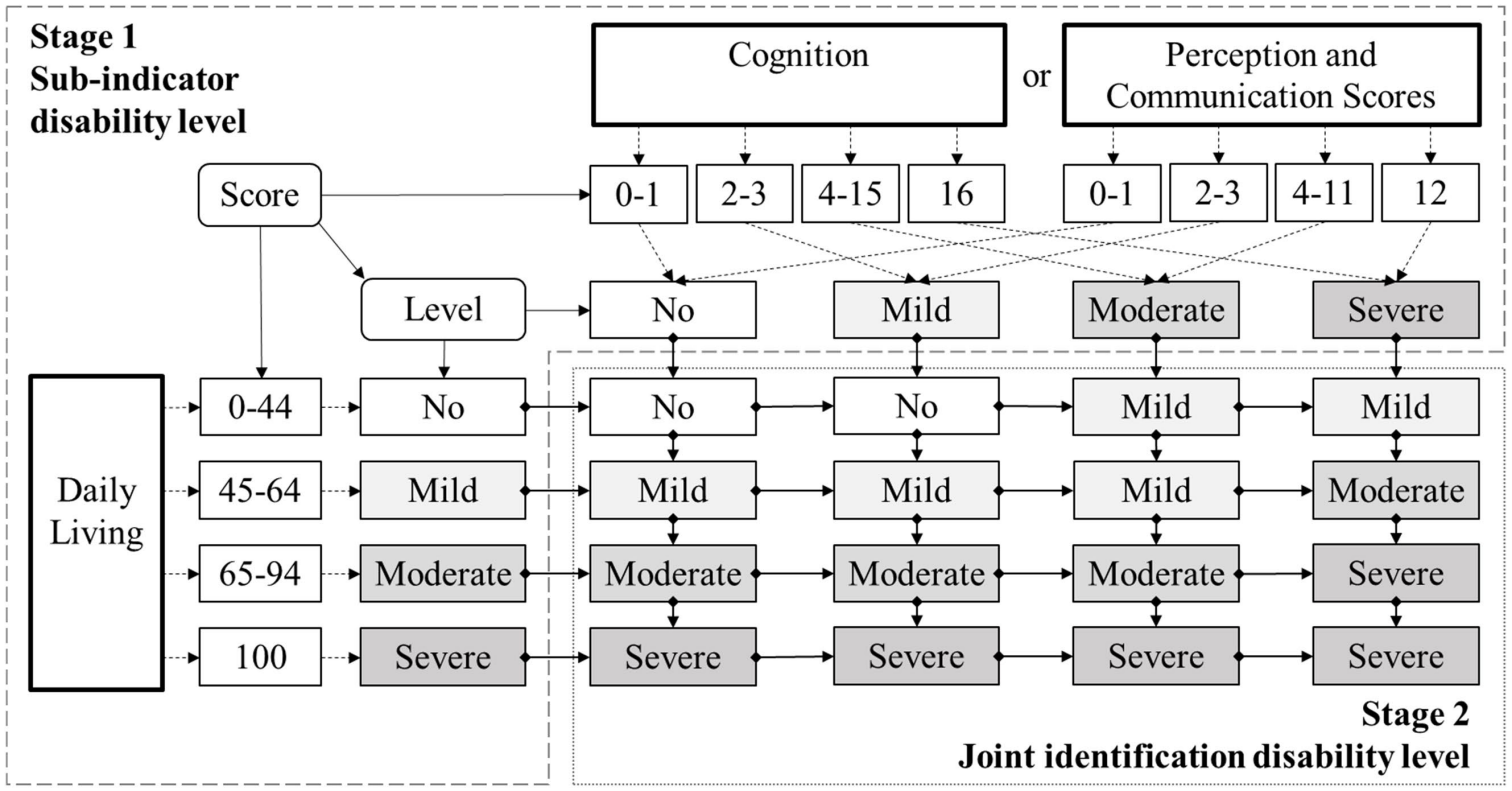
The multi-dimensional joint identification process of disability.

Specific to the assessment process (see Figure 1), first, three abilities of daily living, cognition, as well as perception and communication would be tested to get specific scores^1^, and the scores would be further categorized into 4 levels of “no disability,” “mild disability,” “moderate disability,” and “severe disability.” Second, the level of daily living would be compared with the level of cognition or perception and communication to obtain a comprehensive disability level, which is also divided into 4 levels from no disability to severe disability (see Appendix Table A2). Only the final level of comprehensive disability would be used as the independent variable.

#### 3.2.2 Dependent variable: multidimensional deprivation

This study applied the Alkire-Foster (A-F) method to measure multidimensional deprivation, which has been widely used in multidimensional identification studies to reveal individual or household characteristics (37, 38).

The A-F method is divided into three main steps. First, define the measurement index of multidimensional deprivation and criteria for deprivation cut-off based on the research topic. Second, identify the deprivation status of the samples in single dimension based on the cutoff and weight of each indicator, and then further identify the multidimensional deprivation according to the cutoff of the multidimensional deprivation index and the weight of each dimension. Third, decompose the multidimensional deprivation index according to different dimensions to calculate the contribution of each dimension to deprivation. From this, the final measurements consist of three items, including: (1) Headcount ratio (H), the percentage of people in multidimensional deprivation; (2) Average share (A), suggesting the average share of the deprivation; (3) Multidimensional deprivation index (M0), the product of headcount ratio and average share. The specific calculations for each item are described below.

Let *n* be the number of samples, *d* be the number of dimensions taken into account, *y_ij_* be the value of sample *i* in the *j*th dimension, *w_j_* be the weight of each dimension and $\sum_{1}^{d}w_{j}=1$ . Further, let *z_j_* denote the deprivation threshold within each dimension while constructing an n×d deprivation matrix *g*. If $y_{\mathrm{ij}}<z_{j}$ , then the sample is deprived in that dimension and $g_{\mathrm{ij}}=1$ . The row vector *g^0^* represents the deprivation vector for a series of samples *i*, and the column vector *c* is the number of deprivation dimensions for a series of samples with $c_{i}=\sum_{1}^{j}w_{\mathrm{ij}}q_{\mathrm{ij}}$ . The identification criterion for the multidimensional deprivation status of a specific sample is denoted as $c_{i}(k)$ . The incidence of deprivation $H(k)=\frac{\sum_{1}^{n}q_{\mathrm{ij}}}{n}$ ; and the average deprivation share $A(k)=\frac{\sum_{1}^{n}c_{i}(k)}{\sum_{1}^{n}q_{\mathrm{ij}}\times d}$ . Thus, the multidimensional deprivation index can ultimately be expressed as:


(1) $$M0=d\times \mu (g(k))=\frac{\sum_{1}^{n}q_{\mathrm{ij}}}{n}\times \frac{\sum_{1}^{n}c_{i}(k)}{\sum_{1}^{n}q_{\mathrm{ij}}\times d}=H(k)\cdot A(k)$$

The M0 , H , and A in Equation 1 are all outcome variables in the descriptive analysis of the overall situation of multidimensional deprivation, whereas the regression analysis will use the absolute value of M0 (*MDI*) in individual level and whether or not the individual is in a state of multidimensional deprivation (*MD*) as judged on the basis of that value as the main dependent variables. When analyzing the mechanisms of the formation of multidimensional deprivation, this paper also used three variables of whether an individual is in a state of deprivation in dimensions of economic conditions (*EC*), subjective well-being (*SWB*) and social participation (*SP*). To further reveal the long-term process of multidimensional deprivation in older adults with disability, we also construct a *Period* variable to analyze the effect on the duration of multidimensional deprivation due to old-age disability.

Overall, the above three dimensions and nine indicators^2^ that jointly constitute multidimensional deprivation index, along with their definitions and cutoffs, are shown in Table 1.

**Table 1:** Dimensions, indicators, weights, and deprivation cutoffs.

Dimensions	Indicators	Criteria for deprivation cut-off	Weights
Economic Condition (EC)	Total personal income	The annual per capita net income of each household is less than 2,300 yuan (criteria for poverty at the national level in China, ≈$320).	1/12
	Family Transfer income	No intra-household transfer of financial support in the last year	1/12
	Daily Consume	Per capita daily consumption expenditure below $2 (criteria from the Asian Development Bank)	1/12
	Debt	Household debt greater than 0	1/12
Subjective well-being (ESB)	Self-assessed health	Self-assessed health status as very poor or poorer (compared to average, good and better)	1/9
	Depression	The individual scored 10 and above in the 10 questions of CES-D, a brief screening measure of depression (0 to 9 usually regarded as not depressed)	1/9
	Life satisfaction	Life satisfaction scores of totally dissatisfied or relatively dissatisfied (compared to average, satisfied and relatively satisfied)	1/9
Social Participation (SP)	Intergenerational communication	Frequency of meeting or communicating with family members less than once a month	1/6
	Activity participation	Did not participate in any form of socialization	1/6

(1) The theoretical basis for adopting equal weights for each dimension is assuming that each dimension is equally important for the old people, and by this way the potential disputes of differentiated weights from methods such as principal component analysis can be avoided. (2) Since family transfer income and debt are not only related to economic conditions but also influenced by subjective concepts and other factors, it is difficult to determine appropriate absolute values or proportional values as cutoffs. Thus, whether the situation occurs is used as the criterion for determination. (3) The cutoffs of intergenerational interaction frequency and activity participation are determined by referring to existing researches (50, 51).

#### 3.2.3 Control variables

Control variables include demographic and socioeconomic characteristics at individual level, structure, perceptions, and economic status at family level, as well as gross domestic product in region level. These variables may influence multidimensional deprivation by individual’s economic status, subjective well-being, and social participation, which thus need to be controlled. Table 2 presents the descriptions and basic statistics for main variables.

**Table 2:** Variable definitions and descriptive statistics.

Variables	Descriptions	Mean	S. D.	Min	Max
Disable	0 to 3 representing no disability, mild disability, moderate disability and severe disability, respectively	0.725	0.673	0	3
Gender	Male = 0; Female = 1	0.491	0.500	0	1
Marriage	Divorced/Widowed/Never Married = 0; Married = 1	0.758	0.428	0	1
Age	Actual age	69.090	7.151	60	118
Education	Illiteracy, accounting for:	30.07%			
	No primary school completion	18.30%			
	Primary school	19.08%			
	Junior high school	10.78%			
	Senior high school	5.16%			
	Junior college	0.73%			
	Bachelor degree	18.85%			
	Master’s degree and above	0.02%			
HukouType	Agricultural hukou, accounting for:	74.93%			
	Non-agricultural hukou	23.51%			
	Unified residential hukou	1.51%			
	No hukou	0.05%			
TotalInc	Gross annual personal income in logarithms	7.668	2.805	0	21
MedIns	Medical insurance, Uninsured = 0; Insured = 1	0.910	0.286	0	1
Pension	Pension, Uninsured = 0; Insured = 1	0.818	0.386	0	1
ChildTogether	Whether living with children, No = 0; Yes = 1	0.271	0.444	0	1
ChildEdu	Average years of schooling of children	8.353	3.769	0	23
ChildInc	Annual per capita income of children in logarithms	1.311	0.690	0	2
GDP	GDP per capita in logarithms	10.558	0.602	9	13

### 3.3 Empirical models

Using panel data and instrumental variables is necessary to address the endogeneity in disability and deprivation. According to the type of the dependent variables, the Logit model, the fixed-effect model and the ordered logit model were used to estimate the effects of disability on the incidence, degree and duration of deprivation, respectively. The basic model is shown in Equation 2, where $y_{\mathrm{it}}$ describes the deprivation status (*MD*), deprivation degree (*MDI*), or deprivation duration (*Period*) of samples in time t . $\text{Disabl}e_{\mathrm{it}}$ is the degree of disability. $\text{Control}s_{i,t}$ is a series of control variables. α and $\varepsilon_{i,t}$ are constant term and random error term. ${\text{Individual}}_{i}$ and ${\text{Year}}_{t}$ are individual and time fixed effects, respectively (there are no such two terms in the random effects model).


(2) $$y_{\mathrm{it}}=\alpha +\beta_{0}\text{Disabl}e_{\mathrm{it}}+\text{θControl}s_{i,t}+{\text{Individual}}_{i}+{\text{Year}}_{t}+\varepsilon_{\mathrm{it}}$$

To avoid the problem of bidirectional causality between disability and multidimensional deprivation status as much as possible, we added “average degree of disability among other older adults in the same city excluding the surveyed samples itself” to the model as an instrumental variable $Z_{\mathrm{it}}$ to reach the final model in Equations 3, 4. In this way, the relevance of the instrumental variable with the sample’s independent variable (disability) and the exogeneity with the dependent variable (multidimensional deprivation) can be ensured.^3^


(3) $$\begin{array}{c} \text{Disabl}e_{\mathrm{it}}=\alpha_{1}+\beta_{1}Z_{\mathrm{it}}+\varepsilon_{\mathrm{it}} \end{array}$$

(4) $$\begin{array}{c} \begin{array}{l} y_{\mathrm{it}}=\beta_{0}\alpha_{1}+\alpha +\beta_{0}\beta_{1}Z_{\mathrm{it}}+\text{θControl}s_{\mathrm{it}}+\beta_{0}\varepsilon_{\mathrm{it}}+\\ {\text{Individual}}_{i}+{\text{Year}}_{i}+\varepsilon_{\mathrm{it}}\; \end{array} \end{array}$$

All estimates were done using software Stata 17.

## 4 Results

### 4.1 Disability identification among the older adults

**Figure 2:**
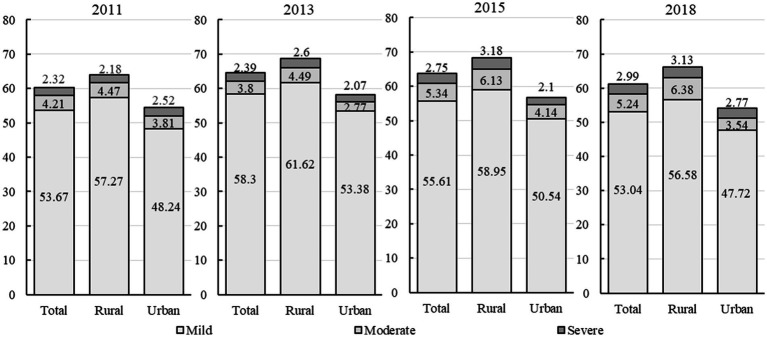
The jointly identified disability among Chinese older adults from 2011 to 2018.

Since this paper used a second-generation assessment tool which jointly identified disability from multidimensional abilities, Figure 2 firstly reports the degree of comprehensive disability among Chinese older adults in urban and rural areas from 2011 to 2018. It can be seen that the overall disability rate of China’s old population had been above 60% for a long time since 2011, and had slightly increased during the surveyed period. Especially the share of moderately and severely disabled seniors in the total number of disabled seniors had increased from 6.5% in 2011 to 8.2% in 2018, implying that as life expectancy increased, the old people would be more likely to face severe disability in later life. However, in terms of urban–rural disparity^4^, the total incidence of disability in rural areas was about 10% higher than that in urban areas. This result means that the long-standing dualistic development model in China indeed led to inequality in health and capacity impairment faced by urban and rural older adults.

In order to show how the disability degree based on above approach differs from disability identified separately from single dimension, Table 3 reports the degree of disability in each of the 3 abilities among Chinese older adults in the same period. If measured only by the ability of daily living, the disability rate would be about 3% lower than the composite disability rate. This result, on the one hand, is basically consistent with the disability rate measured by Gong et al. (34), which can demonstrate the reliability of the sample and data used in this paper. On the other hand, it suggests that a measure only by daily living may ignore some older adults who have normal somatic functioning but are in need of care due to deficits in cognitive and perceptual abilities. If measured in terms of cognitive and perceptual abilities, nearly all older adults are faced with varying degrees of impairments in cognition, perception and communication abilities, which may lead to an overestimation of the severity of disability problems and the actual need for care.

**Table 3:** Incidence of disability among the older adults in urban and rural areas (%).

Year	Degree	Ability								
		Daily living			Cognition			Perception and Communication		
		Total	Rural	Urban	Total	Rural	Urban	Total	Rural	Urban
2011	Total	57.19	60.13	52.76	99.97	99.98	99.96	98.00	98.65	97.04
	Mild	52.22	55.42	47.39	90.80	88.32	94.56	91.17	90.57	92.08
	Moderate	3.32	3.31	3.33	6.94	8.84	4.07	6.64	7.76	4.96
	Severe	1.65	1.40	2.04	2.23	2.82	1.33	0.19	0.32	0.00
2013	Total	61.92	65.67	56.37	100.00	100.00	99.99	96.70	97.44	95.60
	Mild	57.50	60.97	52.36	90.30	87.66	94.20	89.94	89.28	90.92
	Moderate	2.91	3.20	2.48	7.29	9.03	4.71	6.22	7.52	4.30
	Severe	1.51	1.50	1.53	2.41	3.31	1.08	0.54	0.64	0.38
2015	Total	60.63	64.45	54.85	99.97	100.00	99.95	97.02	98.01	95.53
	Mild	55.61	58.88	50.65	86.28	82.83	91.52	91.05	90.83	91.38
	Moderate	3.40	3.81	2.78	9.21	11.49	5.76	5.42	6.54	3.72
	Severe	1.62	1.76	1.42	4.48	5.68	2.67	0.55	0.64	0.43
2018	Total	58.55	62.56	52.55	99.99	100.00	99.97	97.22	98.12	95.89
	Mild	52.77	56.25	47.56	87.10	83.82	92.03	92.24	92.11	92.43
	Moderate	3.70	4.20	2.96	9.07	11.37	5.62	4.89	5.90	3.38
	Severe	2.08	2.11	2.03	3.82	4.81	2.32	0.09	0.11	0.08

Overall, the above results justify the use of the joint identification of disablement, by which way a more accurate estimate of multiple capability impairment can be given.

### 4.2 Measurement and decomposition of multidimensional deprivation

#### 4.2.1 Multidimensional deprivation among the older adults

Since different cutoffs ( k in Equation 1) affect the recognition results of multidimensional deprivations, Table 4 presents the headcount ratio (H), average share (A), multidimensional deprivation index (M_0_), respectively.

**Table 4:** The headcount ratio, average share and MDI among older adults, 2011–2018.

Year	k=1			k=2		
	Total	Rural	Urban	Total	Rural	Urban
Headcount ratio (H)						
2011	0.622	0.685	0.527	0.067	0.083	0.043
2013	0.518	0.602	0.391	0.045	0.057	0.027
2015	0.535	0.607	0.423	0.036	0.045	0.022
2018	0.427	0.497	0.319	0.024	0.032	0.011
Average share (A)						
2011	0.494	0.504	0.471	0.746	0.747	0.721
2013	0.471	0.477	0.455	0.733	0.737	0.741
2015	0.465	0.474	0.447	0.750	0.733	0.727
2018	0.452	0.459	0.436	0.708	0.719	0.636
Multidimensional Deprivation Index (M0)						
2011	0.307	0.345	0.248	0.050	0.062	0.031
2013	0.244	0.287	0.178	0.033	0.042	0.020
2015	0.249	0.288	0.189	0.027	0.033	0.016
2018	0.193	0.228	0.139	0.017	0.023	0.007

The headcount ratio at k=3 is extremely low, especially from 2013 to 2018 when the main indicator is 0, so it is not reported here.

It can be seen that regardless of k , the overall headcount ratio, the average share and the index of multidimensional deprivation all decreased from 2011 to 2018. This means that the multidimensional deprivation of Chinese older adults has declined significantly over recent years. However, as the criterion for identifying deprivation increases ( k=2 ), the incidence and depth of multidimensional deprivation decreased while the average share of deprivation expanded. It is also noteworthy that multidimensional deprivation rather worsened in 2015 compared to 2013 when k=1 , whereas this was not the case when k=2 . This implies that when deprivation is measured from a single dimension, short-term fluctuation in that dimension may lead to the recurrence of exit-return to deprivation, thus affecting the stability of deprivation identification. It can thus demonstrate that deprivation identified from a multidimensional perspective is more stable.

**Figure 3:**
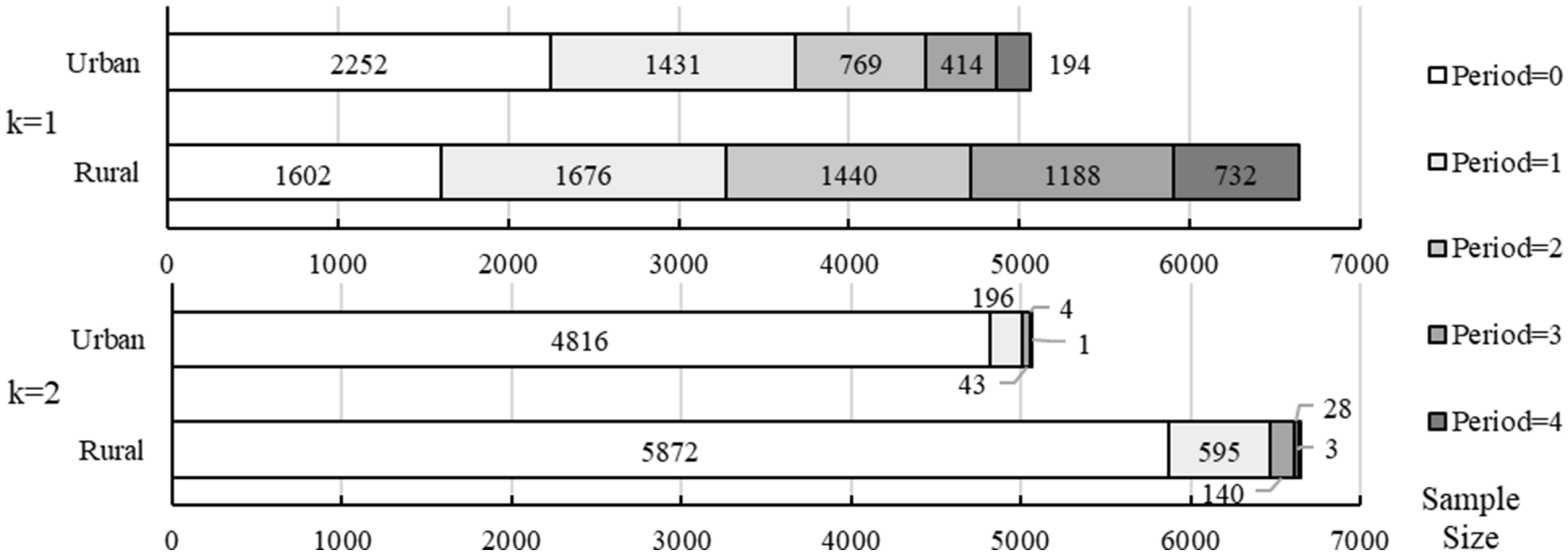
The duration of multidimensional deprivation at different cutoffs.

Figure 3 further reports the sample sizes at different duration of multidimensional deprivation. If k=1 , more than 2/3 of the deprived population were in short-term deprivation of 1 or 2 periods, while the remaining 1/3 were in long-term deprivation more than 4 years. If, only about 9% of the samples were in short-term deprivation, and the samples in long-term deprivation were almost negligible.

In addition, consistent with the urban–rural disparities in disability degree, older adults in rural areas also faced more severe multidimensional deprivation. Specifically, rural areas had a significantly higher headcount ratio, average share, multidimensional deprivation index, and longer deprivation duration than urban areas regardless of the cutoff. And the reduction of headcount ratio in rural areas over time was also lower than in urban areas. As a result, the urban–rural heterogeneity should also be focused on in latter analysis of the impact of disability on multidimensional deprivation.

#### 4.2.2 Decomposition of multidimensional deprivation

In order to further identify the leading cause of multidimensional deprivation, this paper decomposed MDI and compared the results of the decomposition with the incidence of deprivation of the sample on each single dimension (see Table 5). Overall, from 2011 to 2018, the contribution of the economic condition and subjective well-being dimensions to multidimensional deprivation decreased from 31.7 to 24.4% and from 32.1 to 29.9% respectively, while the contribution of the social participation dimension increased from 36.1 to 45.6%.

**Table 5:** Multidimensional deprivation decomposition and unidimensional deprivation rate.

Dimensions	Indicators	Percentage contribution of multidimensional deprivation (%)				Unidimensional deprivation rate (%)			
		2011	2013	2015	2018	2011	2013	2015	2018
Economic Condition (EC)	Total personal income	8.90	11.50	12.60	6.90	43.03	49.08	55.29	24.82
	Family Transfer income	12.60	10.30	9.60	10.10	68.54	48.46	44.71	44.90
	Daily Consume	5.20	7.70	5.10	4.30	24.11	31.41	20.28	14.59
	Debt	5.00	3.40	2.70	3.10	22.35	13.89	10.85	11.25
Subject Well-being (SWB)	Self-assessed health	15.30	16.00	16.10	12.60	56.14	53.57	54.41	32.22
	Depression	11.60	8.10	10.30	12.10	40.33	24.46	31.50	33.13
	Life satisfaction	5.20	4.70	3.30	5.20	15.79	11.83	8.23	11.08
Social Participation (SP)	Intergenerational communication	10.80	12.00	12.40	15.50	21.39	20.19	21.31	22.58
	Activity participation	25.30	26.20	27.80	30.10	52.74	46.48	51.36	52.13

Specifically, family transfer income, self-assessed health, and activity participation were the indicators with higher contribution to MDI and incidence of unidimensional deprivation, and more than half of older adults were under deprivation in the above 3 aspects in 2011. Although the deprivation incidence and contribution of family transfer and self-assessed health indicators had slightly declined over time, the deprivation in the activity participation were even worse, while the intergenerational communication indicator in social participation dimension also shared a growing contribution to multidimensional deprivation. It can be proved that the inclusion of social participation dimension in this paper is crucial.

### 4.3 Impact of disability on endogenous functional deprivation

#### 4.3.1 Baseline regression

As related studies generally identified 1/3 of the total indicators as the multidimensional deprivation cutoff (37), this paper used MDI value in individual level when k=1 to get the outcome variable. Panel A in Table 6 reports the results of the regression of disability on the incidence of multidimensional deprivation (*MD*), the value of multidimensional deprivation index (*MDI*), as well as the deprivation duration (*Period*).

**Table 6:** Baseline regression.

Panel A	*MD*	*MDI*	*Period*	*EC*	*SWB*	*SP*
	(1)	(2)	(3-1)	(4)	(5)	(6)
*Disable*	0.538^***^	0.020^***^	0.293^***^	0.096^***^	0.626^***^	0.418^***^
	(0.027)	(0.002)	(0.023)	(0.024)	(0.027)	(0.026)
Control variables	Controlled					
*N*	30,991	30,991	32,836	32,836	32,836	32,836
(3-2)	*Period* = 0	*Period* = 1	*Period* = 2	*Period* = 3	*Period* = 4	
*Disable*	−0.048^***^	−0.015^***^	0.009^***^	0.026^***^	0.028^***^	
(Marginal Effect)	(0.004)	(0.001)	(0.001)	(0.002)	(0.002)	

Panel B	*MD*	*MDI*	*Period*	*EC*	*SWB*	*SP*
	(1)	(2)	(3)	(4)	(5)	(6)
Stage 1	0.494^***^	0.650^***^	0.521^***^	0.642^***^	0.521^***^	0.521^***^
*ADisable*	(0.033)	(0.039)	(0.027)	(0.104)	(0.034)	(0.034)
Control variables	Controlled					
*N*	30,991	30,991	32,836	32,836	32,836	32,836
Stage 2	0.542^***^	0.026^***^	0.713^***^	0.521^***^	0.686^***^	0.496^***^
*Disable*	(0.127)	(0.008)	(0.103)	(0.034)	(0.110)	(0.117)
Control variables	Control					
*N*	30,991	30,991	32,836	32,836	32,836	32,836

***p < 0.01. Robust standard errors in parentheses.

Model 1 presents the results of the correlation coefficients from the panel random effect estimation, suggesting that the higher level of disability in older adults significantly increased the probability of being multidimensional deprived. The fixed effect estimation in Model 2 indicates that for every 1-level increasing in disability, the deprivation index of older adults increased by 2%. Models 3–1 reports the effects of disability on the duration of multidimensional deprivation, which show that the level of disability was significantly and positively associated with the duration of deprivation. The marginal effects of model (3–2) were further reported below panel A, and the results showed that for the non-deprived older group, the probability of being in non-deprivation sharply dropped as the level of disability increased. However, for older adults who were already trapped in deprivation, an increase in the degree of disability prolonged the duration of their deprivation. The above results are all significant at the 1% level, and validate the central question of this paper, which is that disability does contribute to the multidimensional deprivation faced by older adults.

To further analyze how the above effects occur, Models 4 to 6 report the impact of disability on the deprivation suffered by the sample in each dimension, respectively. The results show that older adults with disability were vulnerable to deprivation due to disability in all 3 dimensions including economic condition, subjective well-being and social participation. Further focusing on the regression coefficients, compared with the economic dimension, the subjective well-being and the social participation dimension were more likely to be deprived due to disability. This suggests that China’s socioeconomic development and anti-deprivation policies may have obvious protective effects on the economic dimension. Especially the Chinese pension system, which has developed rapidly in the past two decades and has a coverage rate of nearly 90%, undoubtedly provides sufficient income protection in old age. However, aspects such as self-assessed health, depression, life satisfaction, intergenerational communication and activities participation, are still vulnerable to the impact of disability. It can be argued that the multidimensional deprivation of older adults caused by disability is formed through all of the 3 dimensions.

Given the bidirectional relationship between disability and deprivation, this study further took “average degree of disability among other older adults in the same city excluding the surveyed samples itself” (*ADisable*) as an instrumental variable to address the potential estimation bias. Panel B in Table 6 reports regression results with the addition of instrumental variables and using two-stage least squares. The results for the 6 models were all statistically significant and consistent with Panel A, further proving the causal relationship of disability on multidimensional deprivation.

#### 4.3.2 Robustness analysis

In order to ensure the reliability of the regression results, this paper used four ways to conduct robustness tests, including replacing dependent variables, adding control variables, using Tobit model for regression, and using samples after propensity score matching (PSM). First, to exclude the above results from being influenced by specific multidimensional deprivation identification strategies, the multidimensional deprivation is re-estimated using k=2 and new explanatory variables are generated for estimation accordingly. Second, to further strip out the deprivation-inducing effects of disability, access to auxiliary (*Auxiliary*) and logarithmic healthcare hours (*LnHT*) were added to the original control variables. Third, due to the large number of 0 values in the dependent variables such as *MDI* and *Period*, the Tobit model for the restricted dependent variable was adopted to have maximum likelihood estimation on left-censored explanatory variables. Finally, propensity score matching (PSM) was used to reduce the impact of sample selectivity bias and confounders on the results. Table 7 shows the regression results under the four methods. The comparison between the results and the baseline regression shows that the estimations in this paper are robust.

**Table 7:** Robustness check.

Method	Variable	*MD*	*MDI*	*Period*	*EC*	*SWB*	*SP*
Adding control variables	*Disable*	0.450^***^	0.018^***^	0.289^***^	0.096^***^	0.519^***^	0.335^***^
		(0.029)	(0.002)	(0.023)	(0.026)	(0.029)	(0.028)
	*Auxiliary*	0.218^***^	0.011^***^	−0.039	0.083^**^	0.259^***^	0.176^***^
		(0.046)	(0.003)	(0.038)	(0.040)	(0.046)	(0.047)
	*LnHT*	0.068^***^	0.001^**^	0.014^*^	−0.019^**^	0.082^***^	0.066^***^
		(0.010)	(0.001)	(0.008)	(0.009)	(0.010)	(0.010)
Replacing dependent variable	*Disable*	0.808^***^	0.020^***^	0.489^***^	0.096^***^	0.626^***^	0.418^***^
		(0.053)	(0.002)	(0.037)	(0.024)	(0.027)	(0.026)
Using Tobit model	*Disable*	0.165^***^	0.040^***^	0.241^***^	0.039^***^	0.158^***^	0.101^***^
		(0.008)	(0.002)	(0.020)	(0.009)	(0.007)	(0.007)
Using PSM sample	*Disable*	0.579^***^	0.022^***^	0.423^***^	0.065^**^	0.704^***^	0.387^***^
		(0.029)	(0.002)	(0.024)	(0.027)	(0.030)	(0.030)

***p < 0.01, **p < 0.05, *p < 0.1.

#### 4.3.3 Urban–rural heterogeneity analysis

Considering the differences in disability and multidimensional deprivation between urban and rural areas, this paper further analyzed whether the impact of disability on multidimensional deprivation also exists urban–rural heterogeneity. Table 8 reports the regression results by urban and rural samples, respectively. Compared to older adults living in rural areas, urban older adults with disability had a slightly higher probability and degree of falling into multidimensional deprivation. Although the economic situation of older adults in urban areas is generally considered better than their rural counterparts, disabled older adults in urban were more likely to suffer from deprivation in economic condition and social participation due to disability.

**Table 8:** Analysis of urban–rural heterogeneity.

Variables			Disable			
	Rural	Urban			Rural	Urban
*MD*	0.089^***^	0.094^***^	*Period*	0	−0.037^***^	−0.068^***^
	(0.006)	(0.007)			(0.004)	(0.008)
*MDI*	0.020^***^	0.021^***^		1	−0.021^***^	0.000
	(0.002)	(0.003)			(0.002)	(0.001)
*EC*	0.010^**^	0.021^***^		2	0.001^*^	0.024^***^
	(0.005)	(0.006)			(0.001)	(0.003)
*SWB*	0.108^***^	0.106^***^		3	0.025^***^	0.026^***^
	(0.006)	(0.007)			(0.003)	(0.003)
*SP*	0.061^***^	0.081^***^		4	0.032^***^	0.018^***^
	(0.005)	(0.007)			(0.004)	(0.002)

***p < 0.01, **p < 0.05, *p < 0.1.

Combined with the marginal effect for the deprivation duration on the right side of the table, although the probability of being deprived for 2 or 3 periods of older adults in urban areas was slightly higher than in rural areas, the probability of being deprived for 4 periods was significantly lower than in rural areas. But the older adults in rural areas had a higher probability of falling from a state of non-deprivation into deprivation due to disability, and were also more likely to be immobilized in chronic deprivation for 4 periods.

## 5 Discussion

Using China as a typical representative, this paper analyzed the impact of disability on multidimensional deprivation based on joint identification of disability and a multidimensional measure of deprivation. The main findings are as follows.

First, the prevalence of disability in Chinese older adults exceeded 60%, with an upward trend especially for the moderate and severe disability groups and a more severe situation in rural areas. Due to the consideration of the impairment of multiple abilities, these results are slightly higher than the estimations in previous studies, while the growing trends and urban–rural heterogeneity of disability are generally consistent (39, 40). It can be deduced that with the development of medical technology and healthcare system, the extending trends of life expectancy and the periods carrying disease in moderately and severely disabled older adults would bring out growing pressure of caregiving and challenges to the worldwide ageing societies.

Second, both the overall headcount ratio and multidimensional deprivation index of older adults in China had decreased significantly. As for the decomposition of multidimensional deprivation and the incidence of unidimensional deprivation, while the contribution and incidence of economic condition and subjective well-being dimensions decreased, the social participation dimension’s contribution to multidimensional deprivation increased. Besides, older adults in rural areas suffer more severe incidence, depth and duration of multidimensional deprivation than their urban counterparts. Although the above results show the achievement of China’s economic development and social policy in poverty alleviation (9), they also suggest that the low awareness of activity participation and the lack of activity facilities in community may be emerging factors contributing to multidimensional deprivation (41, 42).

Third, the disability indeed led to multidimensional deprivation among older adults, with higher levels of disability associated with a higher probability, deeper degree, and longer duration of deprivation, which are consistent with findings of existing research (43). In terms of the mechanisms by which the above effects were produced, disability caused significant deprivation in all three dimensions, but the effects on subjective well-being and social participation were greater than on economic dimensions. Further combined with the theory and reality, it is the adverse outcomes of disability such as decreased income, aggravated depression, reduced life satisfaction and health confidence, as well as diminished engagement in activities (44–47), that caused a multidimensional deprivation in the older adults.

Fourth, as the urban–rural disparities in disability and multidimensional deprivation, the effects of disability on multidimensional deprivation also shared a heterogeneity. For one thing, although urban areas are usually considered to have more resources and opportunities than the rural, urban disabled older adults are more likely to be exposed to economic deprivation and social participation deprivation, which aligns with a Tunisian study on multidimensional vulnerability in households (48). The possible reasons may be that influenced by the traditional filial piety culture, rural older adults may rely on the informal care from family members to a larger extent after they become disable (49), and thus the deterioration in economic condition and intergenerational interaction is relatively small. Comparatively, older adults in urban areas may rely more on formal care, so the decline in intergenerational interaction is relatively greater. Meanwhile, the much higher medical care costs in urban areas further exacerbate the economic deprivation faced by disabled people in urban areas.

For another, the increased disability of older adults in rural areas makes them vulnerable to falling into deprivation from non-deprivation and remaining in chronic deprivation more than 5 years. The possible reason may be that since the entry threshold of the minimum living allowance and other subsidy policies in urban areas is much higher than rural areas, it may take some time for urban disabled older adults to receive compensation. However, due to the better medical resources, social policies, and other opportunities in urban areas, the probability of falling into long-term poverty for urban old people after disability is lower, while the probability for rural older adults is higher.

## 6 Conclusion

Considering that disability and multidimensional deprivation in ageing process are common problems faced by global societies, the typical experience of China can shed light on the improvement of healthcare system and social governance for both developing and developed countries. The potential policy implications are as follows.

First, more effective assessment tools and social policies should be implemented to accurately identify the degree of disability and multidimensional deprivation of older adults, so as to adequately address the diverse needs of the disabled and deprived. For developed countries with more serious problems of ageing and disability, the application of second- or third- generation disability assessment tools considering multiple capabilities in practice should be strengthened. Their advanced development level can provide financial support for ensuring the comprehensive capabilities of the old population. As developing countries facing more severe poverty issues, they may draw lessons from China’s experience, such as incorporating poverty types—like expenditure-based and illness-induced poverty—in poverty identification, and implementing dynamic monitoring of marginalized groups prone to poverty recurrence. The above measures highlight the significance of multiple dimensions and duration in poverty governance.

Second, considering the increasing contribution of the social participation dimension to the level of deprivation, there should be a shift in the relative neglectof social participation in current governance practices. On the one hand, the role of traditional Chinese filial piety culture should be further promoted in building an age-friendly society. On the other hand, relevant facilities (especially in rural areas) and opportunities should be improved to promote the activity participation of the old people. By these ways, the sense of deprivation among the old population can reduced, and their integration into society can be promoted.

Third, since the occurrence of disability will aggravate the multidimensional deprivation suffered by older adults, social protection that can achieve synergistic management of the two issues should be adopted. For example, the economic benefit standard provided by the Long-term Care Insurance should be raised, so as to reduce the economic risks that may be caused by disability. Meanwhile, relevant service for mental health care, functional recovery and social participation can be added to the long-term care service items, thus to avoid deprivation on a larger scale.

Fourth, considering the urban–rural heterogeneity of deprivation resulting from disability, more targeted governance approaches should be adopted. For rural areas with a relatively lower economic level, while strengthening the detection of short-term deprivation of the old people, the problem of long-term deprivation should be addressed through more inclusive and adequate social policies. For urban areas with better economic conditions but more complex social risks and changes in cultural concepts, attention should be paid to the significant differences among the old population in economic capabilities and social participation, so as to prevent some of them from facing greater deprivation due to the untimely policy protection. The above practice is particularly important for developing countries as the development gap will exist within them for a long time.

In conclusion, this study still has limitation at least in the following aspects. Although the CHARLS data is generally representative, influenced by the development process of relevant institution, some *older adults* in rural areas may have been excluded from the sampling frame because they have not been officially registered. Meanwhile, some *older adults* residing in nursing homes or welfare institutions may not have been included in the survey. In fact, these groups may have more serious deprivation or health problems, which may lead to a potential underestimation of the disability and deprivation.

In addition, this study will be expanded from two aspects in the future. First, this study controlled for a series of socioeconomic status variables when estimating the net benefit of disability on multidimensional deprivation. However, since the SES variables not only have direct impact on multidimensional deprivation, but also may further exert indirect influence on multidimensional deprivation through their interaction with disability. Thus, the above mechanisms should be further distinguished to obtain the direct and indirect effect of disability on multidimensional deprivation. Second, the data used in this paper spanned up to 2018, while the pandemic clearly since 2019 obviously has had a dramatic and complex impact on the health, functioning, and broader socioeconomic status of older adults. Against this backdrop, whether the incidence and degree of disability and deprivation in old age exacerbated? Whether the impact of disability on deprivation changed? These questions remain subsequent research to further deepen our understanding of disability and deprivation.

## Data Availability

Publicly available datasets were analyzed in this study. This data can be found here: https://charls.pku.edu.cn/.

## Footnotes

^1^Appendix Table A1 gives the specific proxy variables in CHARLS questionnaire and the detailed scores of 3 abilities.

^2^There are three points that need to be clarified regarding dimensions and indicators. First, the education dimension raised by the United Nations (UNDP, 2023) is excluded here, because as a measure of opportunity equity and competence, education has limited representation of the current situation in old age, and is usually not the main means to improve the old-age welfare. Second, indicators such as self-assessed health, depression, and life satisfaction are used in subjective well-being dimension, which are affected by disability but do not have a direct impact on disability, so as to eliminate potential bidirectional causality and endogeneity problems. However, indicators such as chronic disease are not included here as they have been regarded as the causes of disability. Third, the dimension of social participation is included as an important goal of active ageing.

^3^The instrumental variable of sample i in period tis constructed as follows: $\frac{\sum_{j=1,j\neq i}^{n}\text{Disabl}e_{\mathrm{jt}-}\text{Disabl}e_{\mathrm{it}}}{n_{u}-1}$. Among them, u is the city where the sample i is located, $n_{u}$ is the total number of samples within the city, and j represents the sample in the city excluding i. By this way, on the one hand, the regional characteristics of the old population in health, disease, function and other aspects the average disability level of other samples in this region have a certain degree of connection with the sample itself, thereby meeting the correlation requirement. On the other hand, the disability levels of other samples are not directly related to the multidimensional deprivation degree of the sample itself, thereby meeting the exogeneity requirement.

^4^The criterion for dividing urban and rural areas here is the geographical location of the sample, rather than the Hukou type control variable. And the classification criteria for the analysis of urban–rural heterogeneity in the following text are the same.
