# Supplementary material for: How does disability contribute to deprivation in ageing process: a multidimensional analysis
Source: Front Public Health. 2025 Jun 19;13:1587613. doi: 10.3389/fpubh.2025.1587613 (PMC12224443; doi:10.3389/fpubh.2025.1587613)
Supplement: Supplementary file 1 [file Table_1.DOCX]

**Appendix**

**Table A1** Dimensions of Abilities in Long-term Care Disability Rating Assessment Standards (LCDRAS) and the corresponding questions in CHARLS questionnaire

| **Abilities** | | **Corresponding questions in the CHARLS questionnaire** |
| --- | --- | --- |
| Daily Living  (100 points) | Eating  (10 points) | Because of health and memory problems, do you have any difficulty with eating, such as cutting up your food? |
|  | Dressing  (10 points) | Because of health and memory problems, do you have any difficulty with dressing? |
|  | Facial and oral hygiene  (5 points) | Do you have difficulty with reaching or extending your arms above shoulder level? |
|  | Urination controlling  (10 points) | Because of health and memory problems, do you have any difficulties with controlling urination and defecation? |
|  | Defecation controlling  (10 points) | Because of health and memory problems, do you have any difficulties with controlling urination and defecation? |
|  | Toilet  (10 points) | Because of health and memory problems, do you have any difficulties with using the toilet, including getting up and down? |
|  | Walking  (15 points) | Do you have difficulty with walking 1 km? |
|  | Indoor transferring  (15 points) | Do you have difficulty with getting up from a chair after sitting for a long period? |
|  | Stair climbing  (10 points) | Do you have difficulty with climbing several flights of stairs without resting? |
|  | Bathing  (5 points) | Because of health and memory problems, do you have any difficulty with bathing or showering? |
| Cognition  (16 points) | Time Orientation  (8 points) | What is the year? |
|  |  | What is the date? |
|  |  | What is the day of the week? |
|  |  | What is the month? |
|  | Memory  (8 points) | How would you rate your memory at the present time? Would you say it is excellent, very good, good, fair or poor? |
|  |  | Please remember three objects (Ball, flag, tree) and repeat them. (Record words which are correctly recalled.) |
| Perception and Communication  (12 points) | Vision  (4 points) | Do you have Vision problem? |
|  |  | Do you usually wear glasses or corrective lenses? |
|  |  | How good is your eyesight for seeing things at a distance, like recognizing a friend from across the street (with glasses or corrective lenses if you wear them)? Would you say your eyesight for seeing things at a distance is excellent, very good, good, fair, or poor? |
|  | Hearing  (4 points) | Is your hearing very good, good, fair, poor, or very poor (with a hearing aid if you normally use it and without if you normally don’t)? Would you say your hearing is excellent, very good, good, fair, or poor? |
|  |  | Do you have Hearing problem? |
|  | Communication  (4 points) | Here is a drawing. Please copy the drawing on this paper. |
|  |  | Because of health and memory problems, do you have any difficulties with making phone calls? |

***Notes*:** In fact, the dimension of cognition ability in LCDRAS also includes “Character Orientation” and “Direction Orientation”, and each of the four orientations equally share 4 points. However, the corresponding questions for “Character Orientation” and “Direction Orientation” in CHARLR questionnaire varies with survey years and cannot construct a suitable variable in panel data. Thus, this paper only uses “Time Orientation” and “Memory” in cognitive ability with 8 points equally, so as to ensure that the total score of cognitive ability does not change the rule in conditional identification.

**Table A2** Two-steps Joint identification of Disability

| Stage 1：Sub-indicator disability level | | | | |
| --- | --- | --- | --- | --- |
|  | No  disability | Mild disability | Moderate disability | Severe disability |
| ADL | 100 Points | 65-95 Points | 45-60 Points | 0-40 Points |
| Cognition | 16 Points | 4-15 Points | 2-3 Points | 0-1 Points |
| Perception and Communication | 12 Points | 4-11 Points | 2-3 Points | 0-1 Points |
| Stage 2：Joint identification disability level | | | | |
| Level of ADL | Level of cognition / perception and communication ability | | | |
|  | No  disability | Mild disability | Moderate disability | Severe disability |
| No disability | Level 0 | Level 0 | Level 1 | Level 1 |
| Mild disability | Level 1 | Level 1 | Level 1 | Level 2 |
| Moderate disability | Level 2 | Level 2 | Level 2 | Level 3 |
| Severe disability | Level 3 | Level 3 | Level 3 | Level 3 |

***Notes*:** (1) Joint identification disability level: (i) Level 0: no disability, (ii) Level 1: mild disability, (iii) Level 2: moderate disability, (iv) Level 3: severe disability; (2) In case of different levels of cognitive and perception and communication ability, the worst one was taken into account.
